# Should Degree Programs in Biomedical and Health Informatics be Dedicated or Integrated? Reflections and Recommendations after more than 40 Years of Medical Informatics Education at TU Braunschweig, including 10 Years of B.Sc. and 15 Years of M.Sc. Integrated Degree Curricula

**DOI:** 10.1007/s10916-017-0761-0

**Published:** 2017-06-24

**Authors:** Reinhold Haux, Michael Marschollek, Klaus-Hendrik Wolf, Ute Zeisberg

**Affiliations:** Peter L. Reichertz Institute for Medical Informatics, University of Braunschweig - Institute of Technology and Hannover Medical School, Muehlenpfordtstr. 23, 38106 Braunschweig, Germany

**Keywords:** Biomedical informatics, Health informatics, Medical informatics, Education

## Abstract

Education in biomedical and health informatics (BMHI) has been established in many countries throughout the world. For degree programs in BMHI we can distinguish between those that are completely stand-alone or dedicated to the discipline vs. those that are integrated within another program. After running integrated degree medical informatics programs at TU Braunschweig for 10 years at the B.Sc. and for 15 years at the M.Sc level, we (1) report about this educational approach, (2) analyze recommendations on, implementations of, and experiences with degree educational programs in BMHI worldwide, (3) summarize our lessons learned with the integrated approach at TU Braunschweig, and (4) suggest an answer to the question, whether degree programs in biomedical and health informatics should be dedicated or integrated. According to our experience at TU Braunschweig and based on our analysis of publications, there is a clear dominance of dedicated degree programs in BMHI. The specialization in medical informatics within a computer science program, as offered at TU Braunschweig, may be a good way of implementing an integrated, informatics-based approach to medical informatics, in particular if a dual degree option can be chosen. The option of curricula leading to double degrees, i.e. in this case to two separate degrees in computer science and in medical informatics might, however, be a better solution.

## Introduction

Medical informatics or biomedical and health informatics (BMHI) university degree programs^1^ have been established in many countries throughout the world during the past decades. Informatics programs have existed for some time [1], starting in the early 1960’s at the Ph.D. level [2] and in the early 1970’s at the B.Sc./M.Sc. levels [3]. In 1999 international recommendations on education in BMHI were developed by the International Medical Informatics Association (IMIA), a non-governmental organization of the World Health Organization [4]. A revised version was approved in 2009, and published in 2010 [5].

In *degree programs* of biomedical and health informatics, leading to a specific degree in this field, BMHI specialists are educated ([5], p. 109, Table 1). These degree programs are usually *dedicated programs* or *stand-alone programs*, where graduates receive an informatics degree [9]. At the University of Braunschweig – Institute of Technology (Technische Universität Braunschweig or, briefly, TU Braunschweig), a different educational approach has been taken. The degree program in medical informatics at TU Braunschweig is an *integrated program* [10], offered as a specialization within computer science programs, where informatics graduates, having specialized in medical informatics, can receive a bachelor and a master of science degree in computer science (in German: Informatik) *and* in medical informatics (in German: Medizinische Informatik). The contents of this integrated program is equivalent to respective dedicated BMHI programs. Whether this integrated approach or whether a dedicated approach is more attractive to students, for graduates and for employers of graduates still remains a question, worth to be discussed.

**Table 1:** Dual degree Bachelor of Science curriculum at TU Braunschweig for computer science students, specializing in medical informatics. CP: ECTS credit points. CPs are based on the latest study regulations. Additional medical informatics courses are offered, too, besides those, explicitly mentioned. For specialization in medical informatics ≥50 CP are needed, coming from either computer science electives in medical informatics or from the minor in medicine

B.Sc. in computer science at TU Braunschweig						
competence area	1st	year	2nd	year	3rd	year
	1st semester	2nd semester	3rd semester	4th semester	5th semester	6th semester
computer science [116-121CP]			*Computer science elective courses for students, specializing in medical informatics:* *introduction to medical informatics [5CP], health information systems A [5CP]* [6]*, medical data management [5CP]* [7]*, biomedical signal and image processing [5CP], as well as seminar [5CP], practical course [5CP]* [8]*, and bachelor thesis [15CP] in medical informatics.*			
mathematics [35CP]						
minor [14-19CP]			*Minor in medicine for students, specializing in medical informatics, with courses:* medicine 1 [5CP], medicine 2, [5CP], and health systems [5CP].			
interdisciplinary qualific.[10CP]						
**Σ** **180CP**	≈30CP	≈30CP	≈30CP	≈30CP	≈30CP	≈30CP

After running this integrated medical informatics degree program at TU Braunschweig for 10 years for the B.Sc. and for 15 years for the M.Sc program we want to

- report about this educational approach at TU Braunschweig (section 2),
- analyze recommendations on, implementations of, and experiences with such educational programs in biomedical and health informatics worldwide during the past 10 years, in particular concerning the pros and cons of dedicated degree programs versus integrated degree programs (section 3),
- summarize our lessons learned with the integrated approach at TU Braunschweig (section 4), and, finally,
- suggest an answer to the question of whether degree programs in biomedical and health informatics should be dedicated or integrated (section 5).

Our report, analysis, summary and suggestion are based on our long-term involvement in designing and running TU Braunschweig’s medical informatics curricula. The corresponding author also brings in his experience in contributing to the design of dedicated medical informatics programs in Austria [11] and in Germany [12], as well as his involvement in international activities on informatics education [13, 14].

## The integrated B.Sc. and M.Sc. medical informatics degree programs at TU Braunschweig

At TU Braunschweig, medical informatics education started in 1975. Until 1988, it was a minor in medicine within the University’s computer science program [10, 15]. Since 1996 it has been offered as an application subject within this computer science program. Application subject designation meant that medical informatics courses became part of both the program in computer science as well as the minor in medicine [16]. In the graduates‘diploma certificate, however, only computer science was mentioned as a qualification. Only by going through our graduates‘transcripts, one can see, whether graduates successfully passed the courses of this application field.

Since the winter semester 2001/02, in accordance with the Bologna Process [17] and by applying the European Credit Transfer and Accumulation System (ECTS, [18]), TU Braunschweig introduced a 2 years master program in computer science, comprising 120 ECTS Credit Points (CP), with first M.Sc. graduates in 2002. Since the winter semester 2005/06 a 3 years bachelor program in computer science was introduced, too, comprising 180 CP, with an initial group of B.Sc. graduates in 2008. From October 2005 onwards, these two programs then replaced the traditional German 5 years Diploma-Program, which had been comparable to an integrated bachelor and master program.

An overview of the B.Sc. and M.Sc. computer science programs and of their medical informatics courses, based on the latest study guides [19, 20], can be found in Tables 1 and 2 . A specialization in medical informatics was given, if students successfully passed courses (incl. thesis) with 50 CP or more for the bachelor program and with 70 CP or more for the master program (until winter semester 2005/06 84 CP). Since 2001 for the master program and since 2005 for the bachelor program, students, specializing in medical informatics, can explicitly mention in their bachelor and master certificates medical informatics as *specialization* in addition to their computer science degree. So, since then students can get a dual degree in computer science and in medical informatics.

**Table 2:** Dual degree Master of Science curriculum at TU Braunschweig for computer science students, specializing in medical informatics. CP: ECTS credit points. CPs are based on the latest study regulations. Additional medical informatics courses are offered, too, besides those, explicitly mentioned. For specialization in medical informatics ≥70 CP are needed, coming from either computer science electives in medical informatics or from the minor in medicine

M.Sc. in computer science at TU Braunschweig				
competence area	1st	year	2nd	year
	1st semester	2nd semester	3rd semester	4th semester
computer science [62-68CP]	*Computer science elective courses for students, specializing in medical informatics: health-enabling technologies A [6CP], health-enabling technologies B [4CP], health information systems B [5CP]* [21]*, as well as seminar [5CP], project work [15CP], and master thesis [30CP] in medical informatics.*			
math. & interdisc. Qualific. [8-10CP]				
minor [14-18CP]	*Minor in medicine for students, specializing in medical informatics, with courses:* clinical specialization modules I and II [5CP each], medical-methodology specialization modules I and II [5CP each].			
Σ 120CP	≈30CP	≈30CP	≈30CP	≈30CP

Studying medical informatics at TU Braunschweig is clearly an informatics-based approach, focussing “on the machine processing of data, information and knowledge in health care and medicine with a strong emphasis on the need for advanced knowledge and skills of BMHI, of workflow, people and organizational aspects, of mathematics, as well as of theoretical, practical and technical informatics/ computer science, especially semantic interoperability, ontology-based software engineering and its relationship with effective and safe data, information and knowledge processing and representation” ([5], p. 111). Because of the dual degree, graduates can explicitly show that they are fully qualified as both computer scientists and medical informaticists.

## Recommendations on, implementations of, and experiences with degree educational programs in biomedical and health informatics

In looking at the international state concerning recommendations on, implementations of, and experiences with degree educational programs in biomedical and health informatics, in particular concerning the pros and cons of dedicated degree programs versus integrated degree programs, a Medline search has been done by the first author on January 2, 2017, 8:30 UTC, considering publications in this area during the last ten years, i.e. from 2007 to 2016, using the query (((medical[Title] OR biomedical[Title] OR health[Title]) AND informatics[Title]) AND education[Title]) AND (“2007/01/01”[Date - Publication]: “3000”[Date - Publication]).

79 articles have been selected by this query. As we were interested in recommendations on, implementations of, and experiences with degree curricula, we excluded

- papers not explicitly dealing with such degree programs (e.g. papers on medical informatics courses or course tracks within educational programs in medicine and in the health sciences),
- synopses of best paper selections for the IMIA Yearbook of Medical Informatics,
- literature surveys on informatics education not reporting in detail on degree curricula, and
- papers concentrating on specific methodological contents (e.g. standards available for learning activities, visual analytics).

In order to identify relevant papers among those, having been selected, we chose the following strategy:

- Title has been read: If unclear, the abstract has to be read.
- Abstract has been read: If unclear or if no abstract was available, the full text has to be read.

In one case, the Medline reference was a pointer to a book chapter. There the titles of the book sections have been read. Among the 79 selected publications, we could identify 16 relevant papers, which have all been read as full text [5, 22–36]. A list of the included and excluded articles can be obtained upon request from the first author.

Most of the selected and relevant papers reported on one or several bachelor or master programs at one university. There were reports of BMHI programs of the University of Victoria, Canada [22, 23, 30, 31], of the Aristotle University of Thessaloniki, Greece [24], of the University of Alabama at Birmingham, USA [25], of the Oregon Health & Science University at Portland, USA [28], of the Walter Sisulu University at Mthatha, South Africa [32], and of Charles University at Prague, Czech Republic [35, 36]. All programs were dedicated degree programs. All papers do not discuss the question of whether degree programs in BMHI should be dedicated or integrated. It has, however, to be mentioned that in the meantime the University of Victoria is offering a double degree option, leading to a master degree in nursing and a master degree in health informatics, offering „nurses who are interested in health information technology to develop graduate level competencies in both Nursing and Health Informatics“[37]. Also related is a combined major in health informatics and computer science at this university [38].

Two papers reported about surveys on BMHI programs, one for China [27], and one for the USA [33]. As far as we could see, all degree programs considered were, once again, dedicated programs. As in the reports above, various aspects of curricula design were discussed. But there was no discussion about whether dedicated or integrated informatics curricula should be preferred.

Three articles contained recommendations for BMHI programs with respect to certain regions or countries, in [26] for Europe, in [29] for the USA, and in [34] for the Arab countries. Again, there was no discussion on dedicated versus integrated degree informatics curricula.

A publication of particular relevance was the revised recommendations of IMIA on education in biomedical and health informatics [5]. From the remaining 15 relevant papers 8 of them referred to and cited either these recommendations [25–27, 29, 33–35] or its first version [31]. Although not explicitly raised there, the recommendations report on both approaches. Recommendations for integrated curricula (including also, but not only, integrated informatics degree programs) can be found in section 4, in particular in section 4.4.1 on courses / course tracks for BMHI specialists. Recommendations for dedicated BMHI degree programs are given in section 5. As section 5 is the section dealing explicitly with degree programs, the recommendations seem to prioritize dedicated rather than integrated degree programs.

## Lessons learned at TU Braunschweig

With the specialization in medical informatics within the B.Sc. and in the M.Sc. computer science programs we could show that such integrated informatics degree programs are possible, at least, if an informatics-based approach to BMHI (recall [5], p. 111) has been taken. As mentioned before, with a dual degree in both computer science and medical informatics graduates can explicitly show that they are fully qualified in both fields. At least within Germany there is already for many years a strong demand for such graduates both in the public as in the private sector, with increasing tendency in the current age of digitization.

At TU Braunschweig this approach was and is promoted as a way to attract students. We also published our integrated program in various publications and in German directories of university programs. Partially this was possible. Partially we got replies that such specialisations will not be included in directories of degree curricula, as our medical informatics B.Sc. and M.Sc. curricula are not regarded as degree programs – although we could prove equivalence.

Table 3 shows the number of graduates of our B.Sc. program, and Table 4 those of the M.Sc. program - both up to 2016. In the B.Sc. program we can see that roughly between 5% and 10% of our computer science students specialize in medical informatics. The numbers are small, and for the M.Sc. program they are even smaller. Also, the numbers of students do not correspond to the number of students in our courses, which are higher and the numbers of students, who wrote their bachelor and their master theses at PLRI, which are also documented in Tables 3 and 4, are higher.

**Table 3:** Number of graduates in the dual degree Bachelor of Science curriculum at TU Braunschweig for computer science with specialization in medical informatics until 2016

year	number of graduates with B.Sc. in computer science	number of bachelor theses in medical informatics at PLRI	number of those graduates, having successfully specialized in medical informatics	number of those graduates, having successfully specialized in medical informatics and having requested to mention this in their B.Sc. certificates	relative number of those graduates, having successfully specialized in medical informatics and having requested to mention this in their B.Sc. certificates
2008	4	1	0	0	0%
2009	32	5	4	4	13%
2010	42	8	2	2	5%
2011	34	4	3	3	9%
2012	52	5	2	2	4%
2013	41	7	5	4	10%
2014	43	13	8	6	14%
2015	37	7	2	0	0%

**Table 4:** Number of graduates in the dual degree Master of Science curriculum at TU Braunschweig for computer science with specialization in medical informatics until 2016

year	number of graduates with M.Sc. in computer science	number of master theses in medical informatics at PLRI, In parentheses those written within the traditional German 5 years Diploma-Program (Diplomarbeiten)	number of those graduates, having successfully specialized in medical informatics	number of those graduates, having successfully specialized in medical informatics and having requested to mention this in their M.Sc. certificates	relative number of those graduates, having successfully specialized in medical informatics and having requested to mention this in their M.Sc. certificates
2002	3	1(1)	0	0	0%
2003	8	7(6)	0	0	0%
2004	2	8(4)	2	1	50%
2005	10	9(8)	1	0	0%
2006	14	1(13)	1	0 or 1 ^a^	0 or 7% ^a^
2007	6	10(9)	0	0	0%
2008	10	15(12)	1	0	0%
2009	9	15(13)	1	1	11%
2010	12	9(6)	2	2	17%
2011	34	13(9)	3	2	6%
2012	48	17(9)	7	6	13%
2013	38	15(11)	3	0	0%
2014	33	3(0)	3	1	3%
2015	38	11(0)	4	1	3%

^a^ Could not be verified.

Another observation was that not all students, specializing in medical informatics, do apply for this specialization to be included in their certificates in order to document their dual degree. When asking these students, we learned that all were aware that they can get such a dual degree, but that they finally decided to solely list the computer science degree on their certificates. These students had concerns that the specialization in medical informatics in their certificates could be a disadvantage if they apply for jobs outside medical informatics.

A positive aspect was that this approach was supported by our faculty colleagues in TU Braunschweig‘s Department of Computer Science. Medical informatics was not regarded as competitor but as a field adding to the attraction of studying computer science at TU Braunschweig.

A similar experience can be reported from talking to students. Having the opportunity to specialize within computer science in medical informatics and to obtain a dual degree was seen positively. Some of the students, specializing in medical informatics, told us that they selected TU Braunschweig as the university to attend specifically because of this dual degree opportunity and because they wanted to study medical informatics jointly with computer science. Another reason cited was that not all medical informatics courses had to be taken, but that students have the freedom to decide from semester to semester whether they want to concentrate on medical informatics or on other areas. This lead to situations where students started their studies without planning to specialize in medical informatics, but then were attracted to this field so that they concentrated their studies there. We could also observe the opposite effect, i.e. students planning to specialize in medical informatics, who later shifted during their studies to other areas.

## Dedicated or Integrated?

According to our experience at TU Braunschweig, and based on our analysis of publications, there is a clear dominance of dedicated degree programs in BMHI. The specialization in medical informatics within a computer science program may nevertheless be a good way of implementing an informatics-based approach to medical informatics education, in particular if a dual degree option can be chosen.

Because of the unexpected negative side effects of dual degrees here (students may not be considered as full computer scientists, when medical informatics is mentioned in their certificates), the option of curricula leading to double degrees, i.e. to two separate degrees in computer science and in medical informatics might turn out to be a better solution. In particular, if a large amount of overlap of courses can be implemented in the respective computer science and medical informatics curricula so that the workload and, with it, the study time for obtaining both degrees is not increased by 200% but rather, for instance, by 125%.

The double degree option can be attractive for both the bachelor and master programs. In particular, for master-level programs in medical informatics then, besides the informatics-based approach to medical informatics, a healthcare-based approach ([5], p. 111) could be additionally implemented, especially for students of medicine, of public health and of nursing.

## Discussion

We are aware that the number of graduates is rather small. On the other hand such low numbers can be found in many other publications on informatics curricula (e.g. [2]). Communicating knowledge about these educational programs is, however, important in order to share experiences, which can be then considered for other educational programs. We also want to mention that the literature search in section 3 is not a systematic review according to the PRISMA statement. As the first author has been strongly involved in the design of curricula and in elaborating international recommendations on education, it was regarded in this case as sufficient that he did this search on his own.

In section 4 we mentioned that some students, specializing in medical informatics, did not apply for to have this specialization listed their degrees, because they were afraid that such a dual degree could be of disadvantage if they apply for jobs outside medical informatics. This concern is somewhat surprising, since graduates from dedicated degree programs in medical informatics appear to find good job opportunities in computer science. In [39], p. 582, a shift from medical informatics to computer science was observed for career paths of medical informatics graduates from Heidelberg/Heilbronn.

We discussed the question of dedicated or integrated degree programs for curricula in BMHI. Here the question can be raised whether for other specializations this question might be similar. At least in comparing the specialization in medical informatics within the computer science programs at TU Braunschweig with other specializations (e.g. embedded systems, robotics) we see differences, as BMHI may more than other specializations be regarded as a separate discipline.

Finally, it has to be mentioned that we concentrated our report on B.Sc. and M.Sc. programs, as here the question of dedicated versus integrated is of relevance. We did not report on our approaches for Ph.D. programs in BMHI and on our experiences with them, because, in our opinion, Ph.D. research within any educational framework is, of necessity, dedicated.

## Footnotes

1
Please note that in this text we will use the terms *biomedical and health informatics* and *medical informatics* synonymously, and that we will also use *BMHI* and *informatics* as short versions for these terms.
